# Assessing the consistency of public human tissue RNA-seq data sets

**DOI:** 10.1093/bib/bbv017

**Published:** 2015-03-30

**Authors:** Frida Danielsson, Tojo James, David Gomez-Cabrero, Mikael Huss

**Keywords:** RNA-seq, public data, meta-analysis, gene expression, clustering

## Abstract

Sequencing-based gene expression methods like RNA-sequencing (RNA-seq) have become increasingly common, but it is often claimed that results obtained in different studies are not comparable owing to the influence of laboratory batch effects, differences in RNA extraction and sequencing library preparation methods and bioinformatics processing pipelines. It would be unfortunate if different experiments were in fact incomparable, as there is great promise in data fusion and meta-analysis applied to sequencing data sets. We therefore compared reported gene expression measurements for ostensibly similar samples (specifically, human brain, heart and kidney samples) in several different RNA-seq studies to assess their overall consistency and to examine the factors contributing most to systematic differences. The same comparisons were also performed after preprocessing all data in a consistent way, eliminating potential bias from bioinformatics pipelines. We conclude that published human tissue RNA-seq expression measurements appear relatively consistent in the sense that samples cluster by tissue rather than laboratory of origin given simple preprocessing transformations. The article is supplemented by a detailed walkthrough with embedded R code and figures.

## Background

**Figure 1.**
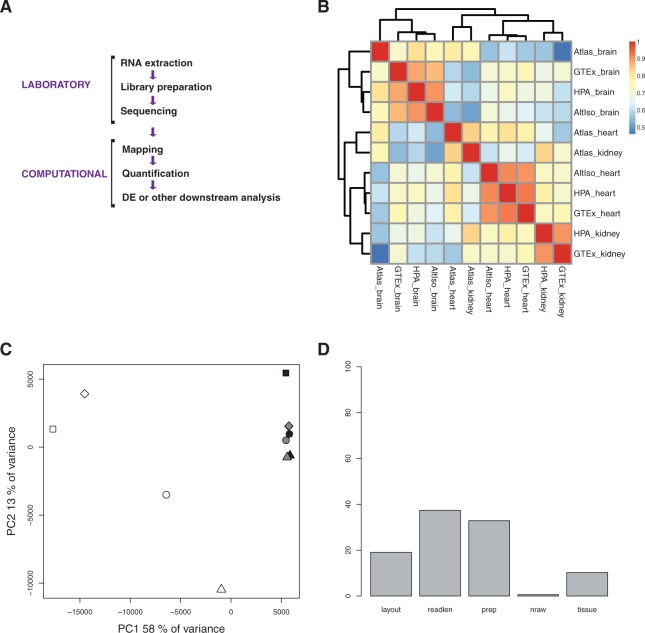
Analyses of the 11 data sets with published precomputed FPKM/RPKM values (*n *= 13,078) for brain, heart and kidney samples from four different studies. (A) Schematic representation of the standard RNA-seq pipeline. (B) Heat map with Spearman correlations between samples. (C) PCA, PC1 and PC2. Tissue types are indicated with white (heart), black (brain) and grey (kidney). Studies are indicated with different symbols; circle (HPA), square (AltIso), diamond (GTex) and arrow head up (Atlas). (D) ANOVA dependence of the laboratory factors layout, read length, preparation, number of raw reads and tissue. tissue. A colour version of this figure is available at BIB online: http://bib.oxfordjournals.org.

Standard RNA-seq experiments begin with RNA extraction, followed by library preparation, amplification and sequencing [1]. Subsequently follows the computational analysis where sequence reads are assembled or mapped to a reference genome and/or transcriptome, quantified and further analyzed to identify biologically interesting expression patterns (Figure 1a).

In recent years, the availability of RNA-seq data sets has dramatically increased, and today several resources are available on the Web where users can download either raw sequence data or precomputed gene expression values, or in some cases query specific genes of interest in tissue-specific contexts. The continuously increasing amount of public expression data promises new opportunities to combine information from different studies, whether in the form of meta-analysis studies (such as genome-wide association studies) [2] or by treating published sample expression profiles as additional samples in one’s own study to add power. For instance, a case-control gene expression study of a disease may benefit immensely from adding previously published data due to the high cost and difficulty of obtaining fresh and relevant samples. Another frequent scenario is that investigators wish to compare their newly produced RNA-seq data with published data as a form of ‘sanity check’—are they similar to the appropriate previously published expression profiles and sufficiently distinct from published profiles that should not be similar?

However, publicly available RNA-seq data sets from different laboratories and studies are sometimes claimed to be incomparable owing to the combined influence of reagent batch effects, differences in RNA extraction protocols, library preparation methods and computational processing (see [3] for a good overview of batch effects in high-throughput biological data), and there are critical issues regarding reproducibility and consistency that need to be considered to make proper use of RNA-seq data. Here, we provide a practical guide for using publicly available RNA-seq data by reviewing the consistency of published expression values measured in Fragments Per Kilobase per Million mapped fragments (FPKM) or Reads Per Kilobase per Million mapped reads (RPKM) from four different studies and evaluating the impact of reprocessing raw data from five studies. In this study, we will focus only on reference-based analysis, leaving aside assembly-based approaches.

The complexity of the RNA-seq pipeline creates many possibilities for bias, and issues regarding the reproducibility of RNA-seq data across laboratories have previously been addressed. For example, one study reported that RNA-seq data are highly consistent as long as the same laboratory protocols and versions of sample preparation and sequencing kits are used [4], and another study reported high intra-platform concordance when testing different protocols across different sequencing platforms for the same samples [5]. However, given that a universal standardization of protocols and equipment across laboratories is not practically possible, comprehensive studies of reproducibility focused on the computational side of the RNA-seq pipeline are needed, to support development of strategies for making RNA-seq data from different studies comparable. A recent study [6] compared different mapping and quantifications pipelines applied to the same (experimental and simulated) data, concluding that the quantification method introduces more variation than the alignment methods.

These studies are useful in that they quantify the variability arising from the sequencing process itself [4], from library preparation type and choice of sequencing platform [3] and from computational processing [6]. To our knowledge, however, no one has yet compared the reproducibility of gene expression profiles from a more practical point of view, where library preparation, sequencing method and computational processing steps are allowed to vary. We therefore set out to do this.

This study focuses on the issue of comparing data sets generated by different laboratories on the same tissue types in samples isolated from different individuals. This is a more practically relevant type of comparison than the ones involving the same samples processed by different labs. Our goals are to examine whether published human tissue expression profiles (from brain, heart and kidney) from different sources are consistent, whether any particular preprocessing transformations are necessary and whether one needs to reprocess all samples in a consistent way from raw sequence FASTQ files instead of working from published FPKM/RPKM values. We also attempt to quantify which factors contribute the most to variability between samples, using principal component analysis (PCA) and analysis of variance (ANOVA). The main condition we use to discuss ‘consistency’ is to see whether the tissue samples (brain, heart and kidney) from several different studies, when subjected to PCA, cluster primarily by tissue rather than by study, or according to some other principle.

Our results indicate that RNA-seq expression profiles in the human tissues studied here appear relatively consistent in the sense that they can readily be made to cluster according to tissue with only light preprocessing.

The best results are obtained by log transformation and application of a batch effect correction method (in this case, ComBat [7]). There appears to be no major benefit of reprocessing the FASTQ files and using Cufflinks [8] to calculate FPKMs rather than just using the reported FPKM or RPKM values from public sources; however, note that this observation may only be valid when samples originate from different tissue types or other biological states, such as in this case.

## Comparing published gene expression levels across studies

### Analysis of untransformed published FPKM/RPKM values

To investigate the consistency of publicly available expression profiles, we first downloaded published precomputed expression levels from four projects involving RNA-seq of human tissue samples: GTEx, Human Protein Atlas, RNA seq Atlas and a study by Wang *et al*., which we call AltIso as an abbreviation of the title of the corresponding paper [9–12] (see Table 1 for information). Depending on study, FPKM or RPKM (the single-end sequencing equivalent of FPKM) values from RNA sequencing of brain (hypothalamus in the case of RNA seq Atlas—we report it as ‘brain’ for simplicity), heart and kidney human tissue samples were analyzed. From the published data sets, genes that were expressed in all samples (FPKM/RPKM > = 0.01) were selected and matched according to ENSEMBL gene IDs, resulting in 13 323 genes for further analysis.

**Table 1.** Characteristics of the public RNA-seq data sets used in this study.

Study	Tissues	FPKM/RPKM	FASTQ	Read type	Total number of raw reads (millions)	RNA extraction method	Reference (PMID)
AltIso	Brain	✓	✓	1 × 28	17.2	PolyA enrichment	18978772
	Heart				20.2		
Evolution of gene expression	Brain		✓	1 × 76	24.5	PolyA enrichment	22012392
	Heart				30.9		
	Kidney				22.5		
GTex	Brain	✓		1 × 76	164	PolyA enrichment	23715323
	Heart				158		
	Kidney				131		
Human Protein Atlas	Brain	✓	✓	2 × 100	28.5	PolyA enrichment	21139605
	Heart				17.7		
	Kidney				16.5		
Illumina BodyMap 2.0	Brain		✓	2 × 75	73.5	PolyA enrichment	22496456
	Heart				82.9		
	Kidney				80.4		
RNA-seq Atas	Hypothalamus	✓	✓	1 × 35,	31.9	rRNA depletion	22345621
	Heart			1 × 50	26.9		
	Kidney				27.2		

Ideally, when visualized using a correlation heat map, principal component plot or another similar technique, the samples should cluster by tissue type rather than study of origin. When plotting the published expression profiles (11 in total) in a correlation heat map, the samples cluster according to their tissue of origin, except for the heart and kidney samples from RNA-seq Atlas (Figure 1b). To avoid the potential problem of irrelevant or noisy genes having an undue influence on correlations, we also plotted the expression profiles in a principal component space. When plotting the first and second components, no ideal separation of tissue types is seen, but the heart samples appear separated from the other tissues in the first component (Figure 1c).

These initial comparisons between the four studies thus indicate that unprocessed published F/RPKM values cannot be considered as reliable reference data, in the sense that it would not be possible to identify the tissue type of an unknown sample based on these values in a correlation heat map or PCA plot. However, the genes contributing the most to the separation in the first three principal components still indicate some degree of tissue specificity. Among the genes with highest loadings in PC1, we find MYL2 (ENSG00000111245), TNNT2 (ENSG00000118194), MB (ENSG00000198125), DES (ENSG00000175084), ACTC1 (ENSG00000159251) and MYH7 (ENSG00000092054), which are all highly upregulated in heart tissue according to the published FPKM/RPKM values. Even though no separation between brain and kidney is visible in the PCA plot, kidney genes [SPP1 (ENSG00000118785), FTL (ENSG00000087086) and ALDOB (ENSG00000136872)] and brain genes [GFAP (ENSG00000131095), CLU (ENSG00000120885) and PLP1 (ENSG00000123560)] are also found among the most contributing genes.

If, instead of looking at gene loadings, we look at correlations between principal component scores and study-dependent experimental factors, we find that the largest variance component (PC1, which explains 58% of the variance) mainly reflects tissue and the second one [PC2, explained variance (henceforth abbreviated e.v.) 13%] mainly correlates with library preparation method and study (Supplementary Figure S1a).

A potential reason for the difficulties in clustering the raw data from different studies is that the F/RPKM distributions could have different distributions. As shown in Supplementary Figure S2a–c, the distributions (log-transformed F/RPKMs are shown for clarity) are different when taken ‘as is’, but one might expect the differences to level after selecting only those 13 323 genes for which an expression value was reported in all four studies. However, the distributions of the expression levels of these common genes (Supplementary Figure S2d–f) still look different between the studies, similar to the original raw data. This picture is consistent across tissues.

Different RNA extraction methods are naturally expected to result in differences in the distribution of RNA molecules. Among the studies in this comparison, RNA Atlas is the only one that used ribosomal RNA depletion (all the others used poly-A enrichment), which leads to a different proportion of mRNA molecules versus noncoding RNA molecules in the sample (the rRNA depleted samples are expected to contain considerably more noncoding RNAs). It appears that the original RNA-Seq Atlas paper calculated RPKM values by dividing counts by the total number of mapped reads (including both non-protein coding and coding genes), which would lead to protein coding genes getting systematically lower RPKM estimates than a poly-A-enriched sample owing to the presence of noncoding RNAs ‘competing’ for the sequencing reads. The published data for RNA-Seq Atlas samples consistently show lower RPKM values than other samples (Supplementary Figure S2a–f, S3).

The fact that distributions greatly differ suggests that a quantile normalization, which attempts to adjust distributions to become more similar, might be able to recover a good clustering by tissue. However, this was not the case for the raw published data (Supplementary Figure S4a–b.)

### Contributions of experimental factors to variability

To quantify how different experimental approaches between the four studies contribute to the overall differences in expression profiles, we used the metadata accompanying the data sets and performed ANOVA analysis after fitting a set of linear models to the data. The factors ‘Layout’ (single or paired end), ‘Read length’ (where in a paired-end case such as 2 × 100, the read length was defined as 200), ‘Preparation’ (RNA extraction method), ‘Total number of raw reads’, ‘Study’ and ‘Tissue’ were included (Table 1). None of the samples included were sequenced using a strand-specific protocol. The estimates of factor contribution to expression variation depend on the order in which factors are specified in the ANOVA linear model. Because we know that the order matters, we set an order that first includes the preparation variability we can account for (layout, read length, RNA extraction method and number of raw reads) and then includes the part of the variability we cannot account for (the study and tissue types). Thus, the contribution of each factor in Figure 1d should be interpreted as the contribution of that factor after all the factors to the left of it in the plot have been accounted for. The contribution of each of the six factors was plotted, excluding the residuals, which include gene-to-gene variation and variance unexplained by the linear models. As shown in Figure 1d, the read length is the factor contributing the most to the variance for the unprocessed published data sets, closely followed by preparation method, layout and only then the tissue type. This indicates that laboratory factors disturb the separation of samples based on tissue type.

### Effects of log transformation

**Figure 2.**
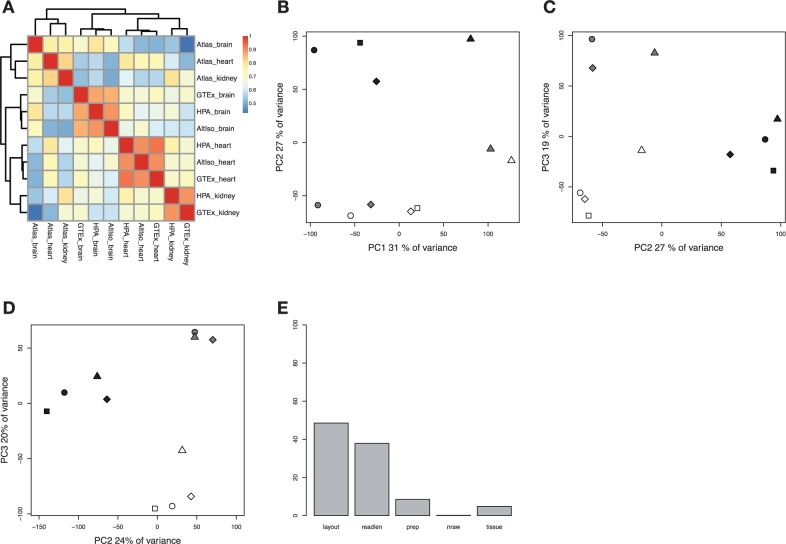
Analysis of log-transformed published precomputed FPKM/RPKM values (*n *= 13,078) for brain, heart and kidney samples from four different studies. In the principal component plots, tissue types are indicated with white (heart), black (brain) and grey (kidney). Studies are indicated with different symbols; circle (HPA), square (AltIso), diamond (GTex) and triangle (Atlas). (A) Heat map with Spearman correlations between samples. (B) PCA, PC1 and PC2. (C) PCA, PC2 and PC3. (D) Cross-validation by PCA excluding the AltIso data with the AltIso data sets projected on top, PC2 and PC3. (E) ANOVA dependence of the laboratory factors layout, read length, preparation, number of raw reads and tissue. A colour version of this figure is available at BIB online: http://bib.oxfordjournals.org.

Logarithmic transformation is known to transform RNA-seq data into a more normally distributed shape with less dependence between the mean and the variance, as well as dampening the effects of extreme outliers [13]. When comparing the 100 most abundant genes (highest FPKM/RPKM), only 9, 22 and 18 of the genes for brain, heart and kidney, respectively, are shared between all studies. This indicates that there is a relatively big group of highly expressed genes that are responsible for the separation of samples of same tissue type, adding complexity to the analysis. As an attempt to stabilize variance and remove bias owing to abundantly transcribed genes, the impact of log transformation of the FPKM/RPKM values was investigated. The published FPKM/RPKM values were log transformed (after addition of a pseudo count of 1) and when plotted in a correlation heat map, the result looks similar as for the non-log-transformed values, except that all three samples from RNA-seq Atlas now cluster together instead of with the correct tissue types (Figure 2a). The effect of the log transformation was also visualized with PCA plots. As shown in Figure 2b and c, the brain samples are separated from the rest of samples when plotting the first (e.v. 31%) versus the second component (e.v. 27%), whereas plotting the second versus the third (e.v. 19%) principal component generates an almost complete separation based on tissue type. To reveal the genes responsible for the separation of tissues, genes with highest absolute loadings were selected. Among the genes contributing most to PC1, the majority shows no consistent tissue specificity according to their published FPKM/RPKM values but a few genes indicate some degree of tissue specificity, like ANKRD1 (ENSG00000148677), CD36 (ENSG00000135218) and TNNT2 (ENSG00000118194) that are upregulated in heart tissue, and FTL (ENSG00000087086) that is upregulated in kidney according to all four studies. Looking at the genes responsible for the separation in the second component, we find several genes with FPKM/RPKM values indicating either brain or heart specificity, for example, CKM (ENSG00000104879), MYL2 (ENSG00000111245), TNN1 (ENSG00000114854), ACTC1 (ENSG00000159251) and MYL3 (ENSG00000160808) (highly expressed in heart tissue) and PLP1 (ENSG00000123560), GFAP (ENSG00000131095), SNAP25 (ENSG00000132639) and MOBP (ENSG00000168314) (highly expressed in brain tissue). These results indicate that there is considerable study-specific bias in the data, responsible for the largest variance component (PC1), that is not removed by log transformation, and that the favorable effect of log transformation, enabling recovery of biological signals that separates the three tissue types from each other, appears in the second and third principal components. Computing correlations of experimental factors to PCs also indicates that the largest variance component is associated with study-specific factors (but not tissue), and that the second largest one mainly reflects tissue (Supplementary Figure S1d). To further confirm that PCs 2 and 3 describe general tissue specificity, we performed a type of cross-validation by first applying PCA to log2-transformed values from all data sets except one (AltIso) and then projecting the AltIso expression profiles onto the obtained components PC2 and PC3. As shown in Figure 2d, the AltIso samples are projected close to the expected clusters, indicating that the tissue-specific information in PC 2 and 3 generalizes to previously unseen samples. To investigate how different experimental approaches contribute to the overall differences between the expression profiles, we again performed ANOVA analysis on the log-transformed values, according to the same procedure as described for the unprocessed values. As shown in Figure 2e, the factors read length and layout are still the most influential factors responsible for the variance between the samples. The influence of the library preparation method appears to have decreased in a relative sense. A possible explanation for this is that there were, as mentioned above, systematic differences between F/RPKM magnitudes between the rRNA-depleted samples (from RNA-Seq Atlas) and poly-A-enriched samples (all other samples; see Supplementary Figure S2A–F, Supplementary Figure S3.) When the F/RPKM values are log transformed, the magnitude differences will be decreased and the importance of the preparation factor for modeling each gene expression estimate will appear smaller relative to other factors.

We also attempted a quantile normalization followed by PCA on the log-transformed values, but were unable to improve on the clustering seen before normalization (Supplementary Figure S4C–D.)

### Correcting systematic study-specific effects

As mentioned before, the three samples derived from the RNA-seq Atlas project are often distinct from the rest of the samples and tend to cluster together rather than with the corresponding tissue type, presumably because the RNA in these samples was purified using rRNA depletion instead of with poly(A) selection of mRNA that was used for all the other samples. The separation of the RNA-seq Atlas samples from the rest exemplifies how different procedures in sample preparation and following sequencing steps can affect the data. In the PCA analysis of log-transformed FPKM values, although the samples cluster better according to tissue type than the unprocessed values, they still appear to be ordered in a consistent pattern according to which study they derive from, indicating again that there are systematic technical effects from all four studies that need to be considered. For instance, the RNA-Seq Atlas samples from each tissue are further away from the tissue-cluster centers than the other samples (Figure 2c); they also have a higher score for principal component 1, which is strongly correlated to library preparation method and read length (see Supplementary Figure S1D).

To adjust for biases introduced in these data sets owing to differences in the library preparation and sequencing for the different studies, we used the ComBat function included in the sva R package for removal of batch effects, only using information on what study the samples derive from [7].

**Figure 3.**
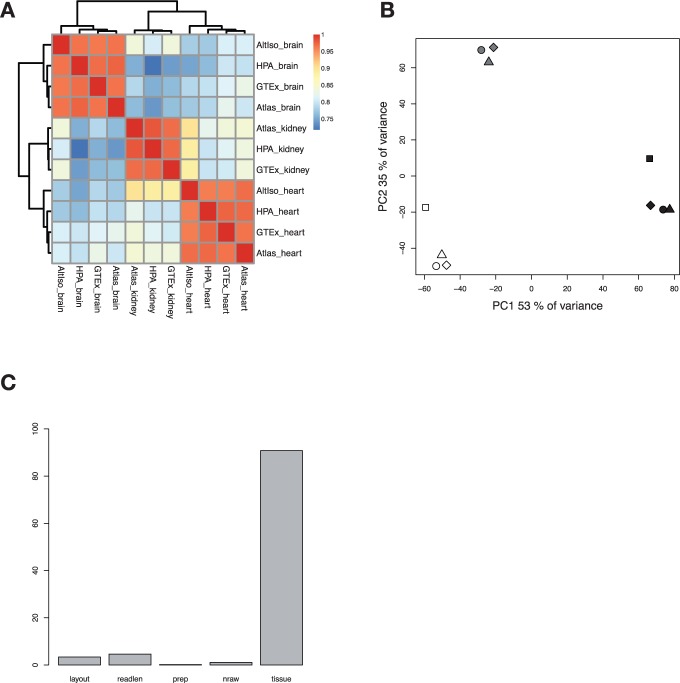
Analysis of precomputed FPKM/RPKM (*n *= 13,078) values for brain, heart and kidney samples from four different studies after removal of batch effects using ComBat. In the principal component plot, tissue types are indicated with white (heart), black (brain) and grey (kidney). Studies are indicated with different symbols; circle (HPA), square (AltIso), diamond (GTex) and triangle (Atlas). (A) Heat map with Spearman correlations between samples. (B) PCA, PC1 and PC2. (C) ANOVA dependence of the laboratory factors layout, read length, preparation, number of raw reads and tissue. A colour version of this figure is available at BIB online: http://bib.oxfordjournals.org.

As shown in Figure 3a, after running ComBat, the samples cluster perfectly according to tissue type when plotted in a correlation heat map, indicating that study-specific effects have now been mitigated. When again performing PCA analyses after the ComBat run, the samples are clearly separated based on tissue type, and according to the previously described ANOVA analysis, tissue type is now responsible for most of the variance between samples apart from residuals that are not included in the plot (Figure 3b and c).

We conclude from the above analyses that published precomputed expression levels for human tissue RNA-seq data from different sources are poorly comparable at a global level and that log transformation and modeling of known batch effects are essential to make the data comparable as consistent reference data. Next, we examined whether the results could be improved by reprocessing the data from raw FASTQ sequence files.

### Effects of reprocessing raw sequence data

**Figure 4.**
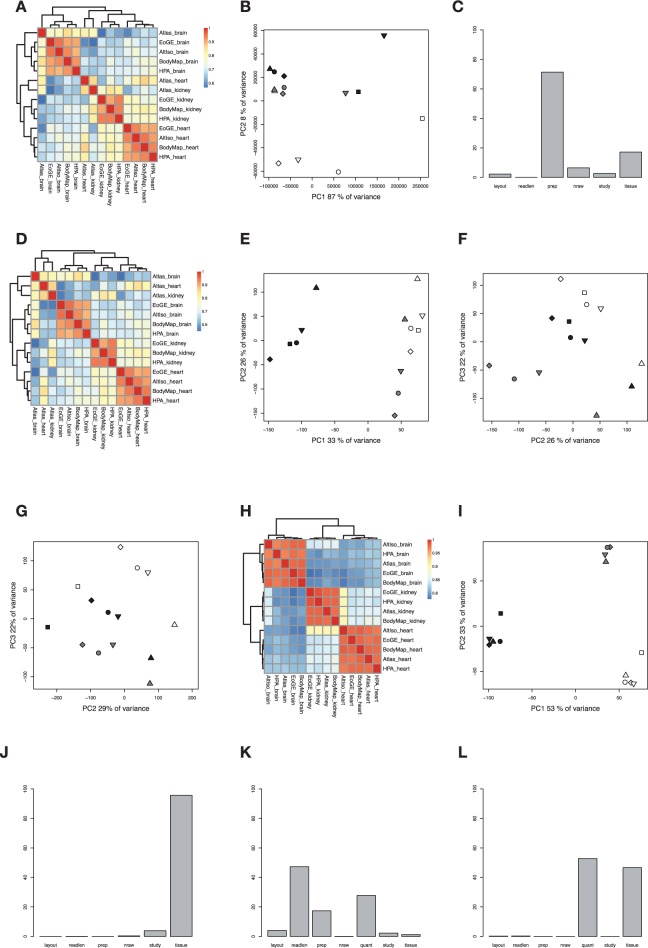
Analysis of Cufflinks FPKM values (*n *= 18,175) after reprocessing from FASTQ files. In the principal component plots, tissue types are indicated with white (heart), black (brain) and grey (kidney). Studies are indicated by different symbols; circle (HPA), square (AltIso), diamond (EoGe), arrow head up (Atlas) and arrow head down (BodyMap). (A) Heat map with Spearman correlations between samples. (B) PCA, PC1 and PC2. (C) ANOVA dependence of the laboratory factors layout, read length, preparation, number of raw reads, study and tissue. (D) Heat map with Spearman correlations between samples after log transformation. (E) PCA with log-transformed data, PC1 and PC2. (F) PCA with log-transformed data, PC2 and PC3. (G) Cross-validation by PCA excluding the AltIso data with the AltIso data sets projected on top, PC2 and PC3. (H) Heat map with Spearman correlations between samples after removal of batch effects using ComBat. (I) PCA after ComBat, PC1 and PC2. (J) ANOVA dependence of the laboratory factors layout, read length, preparation, number of raw reads, study and tissue, after ComBat. (K) ANOVA dependence of the laboratory factors layout, read length, preparation, number of raw reads, quantification method, study and tissue, after joining precomputed and recomputed F/RPKM values together. (L) ANOVA dependence of the laboratory factors layout, read length, preparation, number of raw reads, quantification method, study and tissue, after joining precomputed and recomputed F/RPKM values together and removal of batch effects using ComBat. A colour version of this figure is available at BIB online: http://bib.oxfordjournals.org

To remove potential bias from the fact that different bioinformatics pipelines were used to calculate published FPKM/RPKM values, we repeated the same analyses as above using FPKM values obtained from reprocessing raw data (FASTQ files) in a consistent way. We used human tissue RNA-seq data from five studies that had published FASTQ files: BodyMap, Evolution of Gene Expression, Human Protein Atlas, RNA-seq Atlas and AltIso [10–12, 14, 15]. (See Table 1 for information). FASTQ files for human brain (hypothalamus in the case of RNA-seq Atlas), heart and kidney samples from each of the five sources were downloaded and mapped to the human genome (GRCh37) using TopHat [16], and FPKM values were calculated using Cufflinks [8]. For the list of FPKM values generated with Cufflinks, all non-protein coding genes and all genes with FPKM ≤0.01 in all samples together were first filtered out, resulting in expression levels for 19 475 genes. When first plotting the FPKM values generated with Cufflinks in a correlation heat map, a similar result as for the corresponding published values is obtained with all samples clustering together according to tissue type, again with exception for the heart and kidney samples from RNA-seq Atlas (Figure 4a). As shown in Figure 4b, plotting the first versus the second principal component also generates a result corresponding to that obtained for the published FPKM/RPKM values with the heart samples separated from brain and kidney samples, but this time a tendency toward a separation of all three tissue types is visible in the second component. PC1 (e.v. 87%) correlates most strongly with preparation method, whereas PC2 (e.v. 8%) correlates with tissue (Supplementary Figure S1b).

The genes with highest loadings in the first three principal components were various mitochondrially encoded genes with relatively high FPKM values (up to hundreds of thousands in FPKM) in all samples, together with a few tissue-specific genes, for example, APOE (ENSG00000130203), ALDOB (ENSG00000136872) and YBX3 (ENSG00000060138) that are highly upregulated in kidney (YBX3 is highly expressed in kidney according to all studies except RNA-seq Atlas where it shows high expression in heart). For details, see accompanying walkthrough. To avoid this type of noise from highly expressed mitochondrially encoded genes, a masking option for mitochondrial genes can be used when running Cufflinks. ANOVA analysis was again performed, and as shown in Figure 4c, the contribution from the factor tissue is higher as compared with the results obtained for the published precomputed data sets even though the preparation (RNA extraction method) is clearly the most important factor for the differences between the expression profiles. According to these results, reprocessing of raw data has the advantage of dampening the effects of the laboratory factors Layout and Read length and increasing the contribution of tissue type on the variance, although not enough to yield a clustering by tissue type in PCA. One way to understand this is that in contrast to the published data, the layout and read length are handled in a consistent way by the bioinformatics pipeline in the reprocessed data, which results in a smaller contribution to variation from these factors.

The impact of log transformation was also investigated for the reprocessed FPKM values, resulting in a similar result as the published values. As shown in Figure 4d, the tissues cluster together except for the samples from RNA-seq Atlas. For the log-transformed reprocessed values, PCA analysis also generates similar results as the published data, but the separation of tissues seen in the second and third principal components is not as successful this time, potentially a consequence of the fact that the reprocessed data sets include >6000 more genes compared with the published data, introducing more variation (Figure 4e and f). When we performed cross-validation in the same way as for the published FPKM/RPKM values, the AltIso samples do not cluster to the expected samples (Figure 4g). In other words, the PCA grouping is not stable to addition of new samples. Perhaps surprisingly, log transformation thus appears less effective in recovering tissue signal in PCs 2 (e.v. 26%) and 3 (e.v. 22%) in reprocessed data than in published data (also cf. Supplementary Figure S1e.) Logarithmic transformation again serves to improve the clustering of tissue types to some extent, but there is still systematic variation in the raw data that remains problematic even after removal of potential bias from differences between bioinformatics pipelines. To overcome the problem with laboratory batch effects in the raw data, we used the ComBat function for removal of known batch effects. As shown in Figure 4h, clustering after running ComBat on log-transformed values is again remarkably successful with all samples clustering completely according to the right tissue of origin. As shown in Figure 4i, a complete tissue separation now appears already when plotting the first versus the second principal components, and according to ANOVA, tissue type is now the factor responsible for the most variance between samples (Figure 4j).

In summary, we conclude that the reprocessing of raw data does not *per se* improve the overall consistency of RNA-seq expression profiles, although it does avoid the loss of genes associated with combining gene identifiers from different annotation systems.

### A joint analysis to quantify pipeline effects on variation

A different way to address the question how much the choice of the bioinformatics pipeline (or quantification method, including mapping software and F/RPKM calculation method) influences the results is to perform ANOVA and PCA correlation analyses as described above but adding the quantification as an additional factor. We cannot do such an analysis for the published data alone, because each study used a different bioinformatics pipeline, and thus the effects of the pipeline would be impossible to distinguish from other factors that varied between the studies. We therefore combined both the published and reprocessed data and performed ANOVA with the new factor ‘quantification’ that represents the mapping and quantification steps. All reprocessed samples were quantified with the Tophat/Cufflinks pipeline, as were the HPA samples in the published data. The ANOVA on untransformed F/RPKM values (Figure 4k) indicates that the quantification method is important compared with most other factors, explaining a large fraction of the variance after the sequencing and library preparation protocols have been accounted for. Notably, after log transformation and batch effect correction (Figure 4l), the quantification method even explains slightly more of the variation than the tissue, whereas all other factors are essentially uninformative.

This result is in apparent conflict with the finding above, namely, that reprocessing data from FASTQ in a consistent way does not improve the clustering of the samples by tissue (which it would be expected to do if different quantification methods introduced systematic bias.) We speculate that the differences in quantification methods contribute to gene-to-gene variation and unspecific noise, but not to systematic variation that would affect samples from separate tissues in different ways. An analysis of the correlations of the first two principal components to the experimental factors (Supplementary Figure S1c, f) indicates that the main directions of variation in the data are not strongly correlated to the quantification method.

## Conclusion

We here present a thorough comparison of public human tissue RNA-seq data sets including both precomputed values and consistently reprocessed data sets for ostensibly similar samples (specifically, human brain, heart and kidney samples). With the findings of this study, we conclude that publicly reported precomputed values for gene expression (FPKM/RPKM) are not comparable at a global level in their untransformed state. However, after log transformation and removal of batch effects, the data show global consistency. Logarithmic transformation alleviates problems in clustering of the three tissue types, but is still not sufficient to enable the most of the variance to be explained by tissue type. The largest variance is explained by known or unknown study-specific effects that will disturb clustering unless they are identified using statistical modeling, and removed before analysis. Today, numerous methods are available for bias detection and removal and in this study, ComBat was successfully used for that purpose, although one of several alternative methods could have been used [17]. Reprocessing of raw data does not contribute to any obvious improvement in consistency of the three tissue types. Apart from the advantage of increasing coverage by avoiding the loss of genes in combining different types of identifiers, and of apparently decreasing the impact from layout and number of raw reads on variance, there is no improvement gained in clustering by reprocessing from the FASTQ format.

There are many potential alternatives to the various RNA-seq data processing steps, but we have confined this study to a relatively standard workflow for the sake of clarity. Potential improvements include alternative methods for variance stabilization transformations (in contrast to the log transformation used in this case) and other normalization methods, for example, GC content correction methods and scaling methods like Trimmed mean of M values. The choice of PCA as the visualization method was made based on its popularity, but it is possible that other methods such as multidimensional scaling, nonnegative matrix factorization or t-distributed stochastic neighborhood embedding could have yielded better results. Additionally, it would be interesting to use a different set of tissues that are more similar in their expression profiles compared with the three tissue types used in this study. Nevertheless, we expect that the findings of this study will play an important role in the continuous efforts to secure the usefulness of public RNA-seq data and fulfill the requirement of reliability in comparisons between data sets generated in different laboratories.

## Supplementary data

Supplementary data are available online at http://bib.oxfordjournals.org/. In addition, a walkthrough of the analyses in the article (as well as many variations not reported in the article in the interest of clarity) with embedded code and figures is available at https://github.com/hussius/publicRNA seqdata.

**Key Points**

- Publicly available data sets with precomputed RNA expression levels are not comparable in their untransformed state in the sense that samples from the same tissues obtained in different experiments do not cluster by tissue.
- Logarithmic transformation improves clustering of samples in principal components 2 and 3, while principal component 1 still seems to be dominated by study-specific factors.
- RNA extraction method, read length and sequencing layout (single-end versus paired-end) contribute strongly to variation between samples.
- Removal of known batch effects is essential for clustering based on tissue type.
- Reprocessing raw data avoids loss of expression information because of gene identifier matching issues but does not serve to improve clustering.

## Funding

Knut and Alice Wallenberg Foundation (to M.H.); EU FP7 STATegra 306000 (to D.G.C.); EU FP7 Affinomics 241481 (to F.D.).

## Supplementary Material

Supplementary Data
