# Supplementary material for: Assessing the consistency of public human tissue RNA-seq data sets
Source: Brief Bioinform. 2015 Mar 30;16(6):941–9. doi: 10.1093/bib/bbv017 (PMC4652619; doi:10.1093/bib/bbv017)
Supplement: Supplementary Data [file supp_16_6_941__index.html]

Assessing the consistency of public human tissue RNA-seq data sets — Assessing the consistency of public human tissue RNA-seq data sets — Supplementary Data 

# Assessing the consistency of public human tissue RNA-seq data sets

## Supplementary Data

files

**Files in this Data Supplement:**

- Supplementary Data - docx file
- Supplementary Data - jpg file
- Supplementary Data - jpg file
- Supplementary Data - jpg file
- Supplementary Data - jpg file
