# Supplementary material for: Assessing the consistency of public human tissue RNA-seq data sets
Source: Brief Bioinform. 2015 Mar 30;16(6):941–9. doi: 10.1093/bib/bbv017 (PMC4652619; doi:10.1093/bib/bbv017)
Supplement: Supplementary Data [file supp_bbv017_New_Microsoft_Office_Word_Document.docx]

**Supplementary Figure legends**

**Figure S1.** Correlations of various study dependent factors with principal components. **(A)** Published F/RPKM values, principal components 1 and 2. N=13078 **(B)** Re-processed data, principal components 1 and 2. N=18175 **(C)** All data joined together, principal components 1 and 2. N=12945 **(D)** log-transformed published F/RPKM values, principal components 1 and 2. N=13078 **(E)** log-transformed re-processed data, principal components 1 and 2. N=18175 **(D)** log-transformed joint data, principal components 1 and 2. N=12945.

**Figure S2.** Density plots showing the distributions of log-transformed FPKM values in the different studies. **(A)** Distribution of FPKM values for the non-merged data for brain samples from the studies AltIso (N=15533), Atlas (N=5530), GTEx (N=18684) and HPA (N=19128). **B)** Distribution of FPKM values for the non-merged data for heart samples from the studies AltIso (N=15533), Atlas (N=5530), GTEx (N=18684) and HPA (N=19128). **C)** Distribution of FPKM values for the non-merged data for kidney samples from the studies Atlas (N=5530), GTEx (N=18684) and HPA (N=19128). **D)** Distribution of FPKM values for the merged data (N=13078) for brain samples from the studies AltIso, Atlas, GTEx and HPA. **E)** Distribution of FPKM values for the merged data (N=13078) for heart samples from the studies AltIso, Atlas, GTEx and HPA. **F)** Distribution of FPKM values for the merged data (N=13078) for kidney samples from the studies Atlas, GTEx and HPA. **G)** Distribution of FPKM values for the re-processed Cufflinks data (N=18175) for brain samples from the studies AltIso, Atlas, BodyMap, EoGE and HPA. **H)** Distribution of FPKM values for the re-processed Cufflinks data (N=18175) for heart samples from the studies AltIso, Atlas, BodyMap, EoGE and HPA. **I)** Distribution of FPKM values for the re-processed Cufflinks data (N=18175) for kidney samples from the studies Atlas, BodyMap, EoGE and HPA.

**Figure S3.**

Box plots showing the distribution of F/RPKM values for the eleven samples in the published data. For clarity, outliers have been removed.

**Figure S4.**

Principal component analysis of published pre-computed FPKM/RPKM values after quantile normalization of raw and log-transformed values (n=13078) for brain, heart and kidney samples from four different studies. Tissue types are indicated with red (heart), blue (brain) and green (kidney). **A)** Principal component plot with quantile normalized F/RPKM values, PC1 and PC2. **B)** Principal component plot with quantile normalized F/RPKM values, PC2 and PC3. **C)** Principal component plot with quantile normalized log-transformed F/RPKM values, PC1 and PC2. **D)** Principal component plot with quantile normalized log-transformed F/RPKM values, PC2 and PC3.
