# Supplementary figures and images for: Assessing the consistency of public human tissue RNA-seq data sets
Source: Brief Bioinform. 2015 Mar 30;16(6):941–9. doi: 10.1093/bib/bbv017 (PMC4652619; doi:10.1093/bib/bbv017)

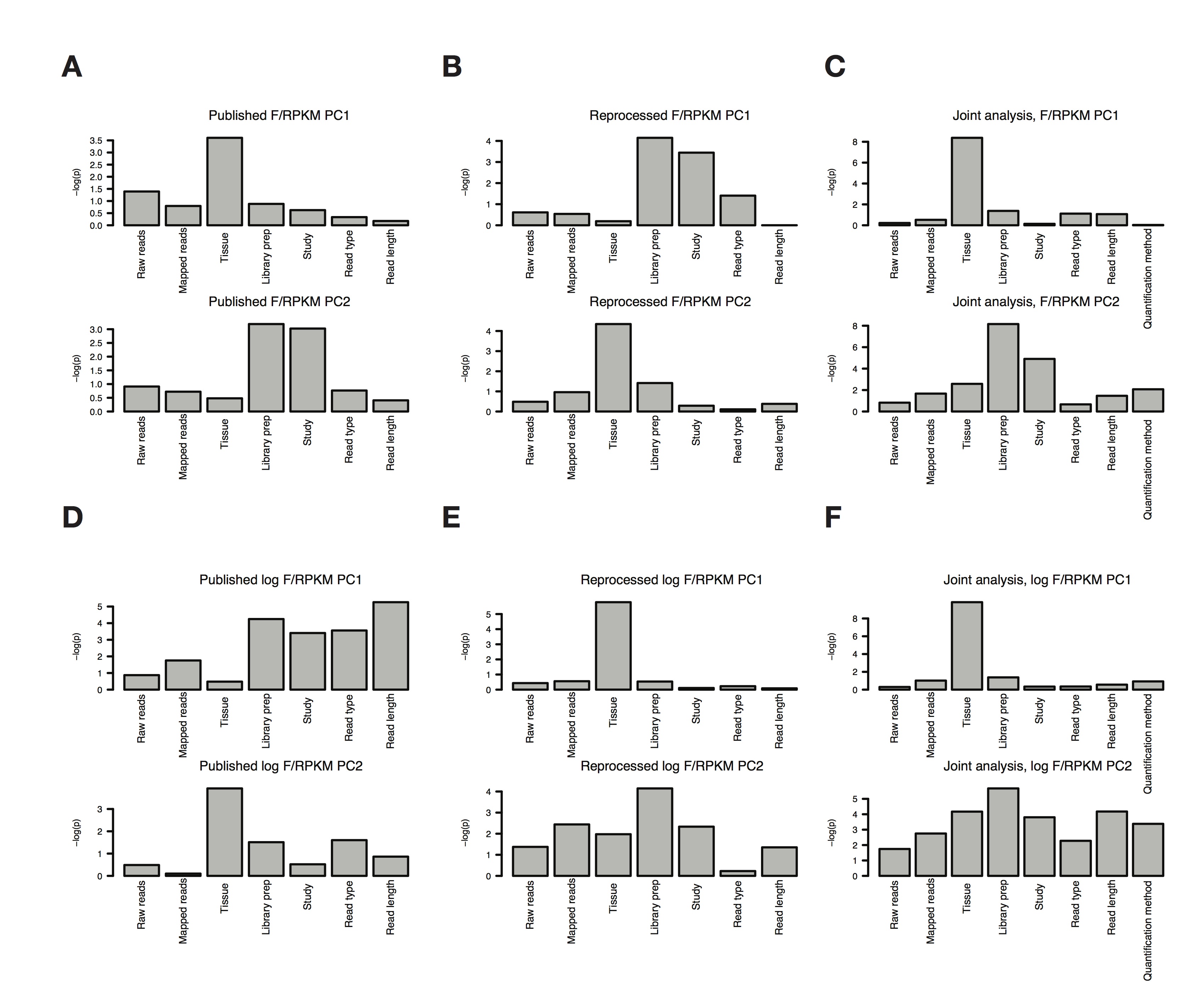

Supplement: Supplementary Data [file supp_bbv017_SupplementaryFigure1.jpg]

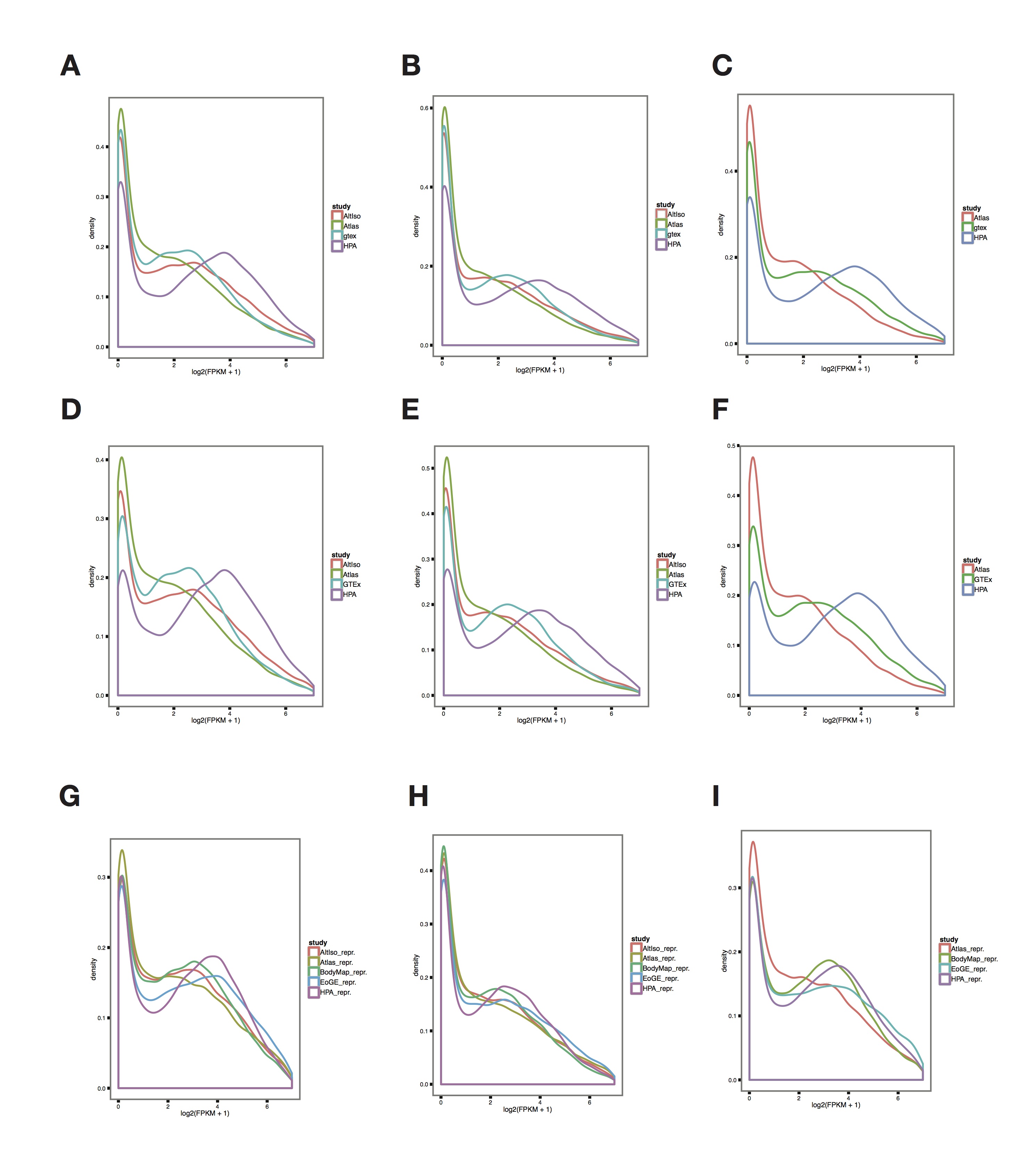

Supplement: Supplementary Data [file supp_bbv017_SupplementaryFigure2.jpg]

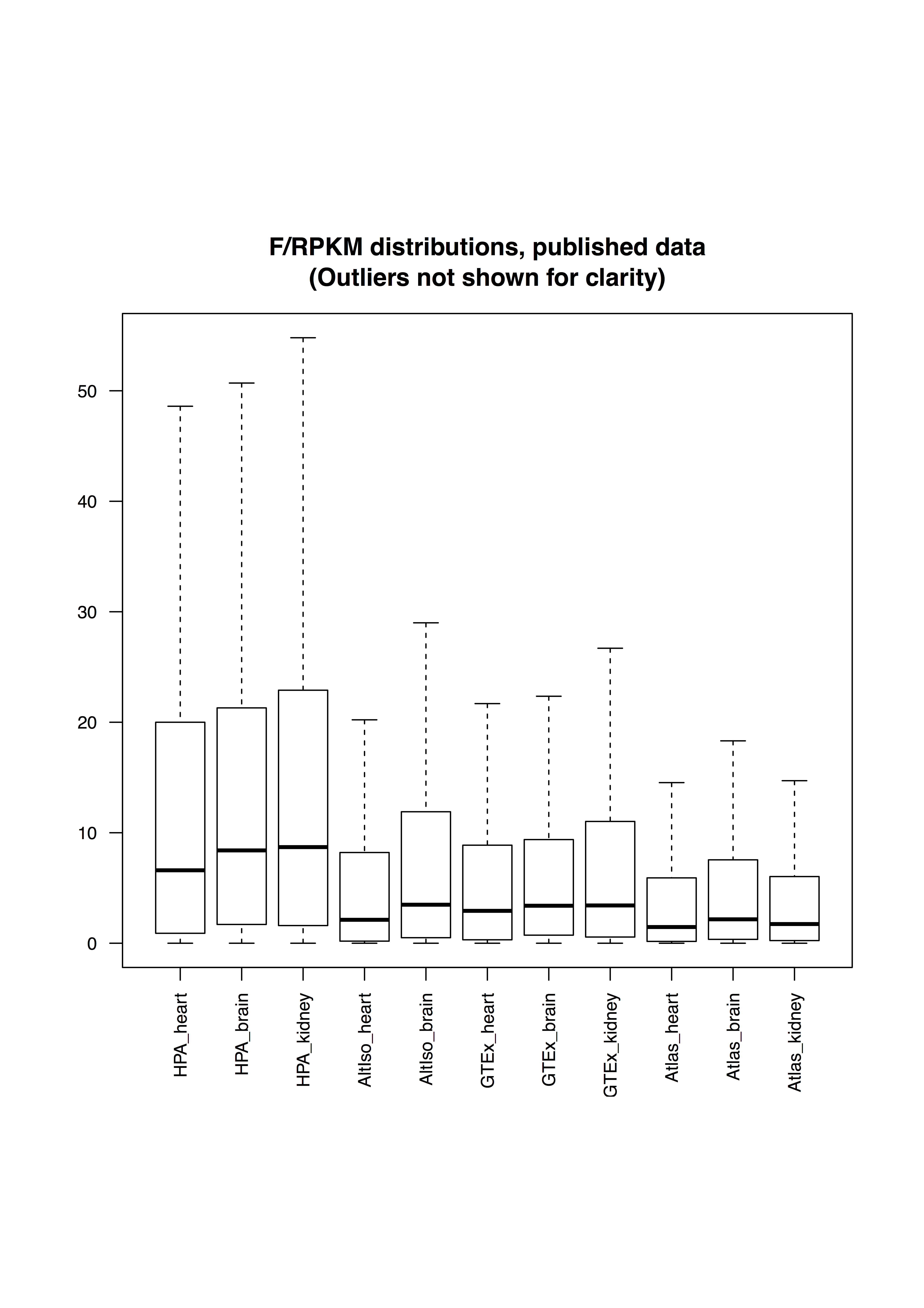

Supplement: Supplementary Data [file supp_bbv017_SupplementaryFigure3.jpg]

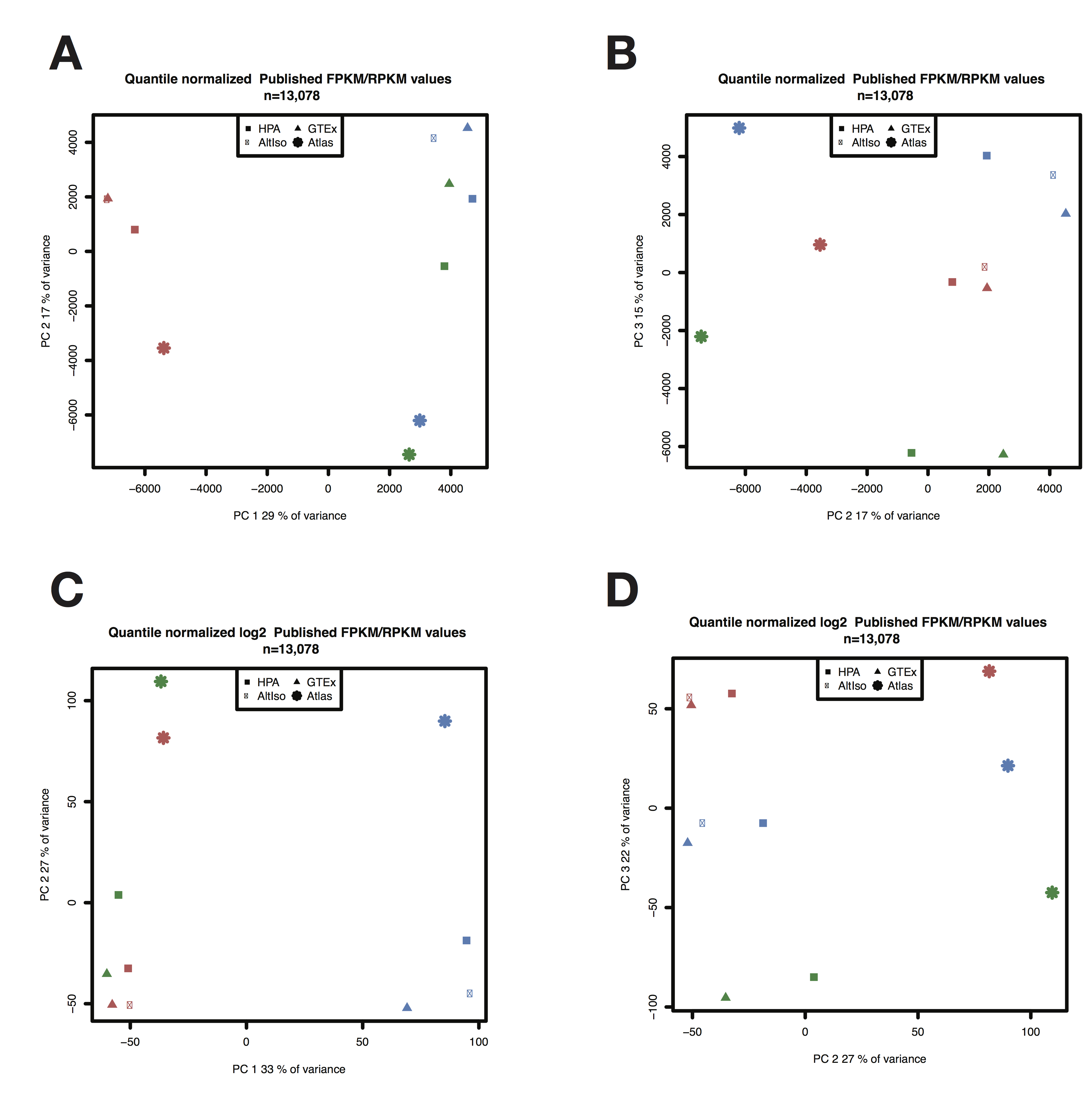

Supplement: Supplementary Data [file supp_bbv017_SupplementaryFigure4.jpg]
